# 4569 Burden of illness in idiopathic pulmonary fibrosis: A real-world cohort

**DOI:** 10.1017/cts.2020.111

**Published:** 2020-07-29

**Authors:** Erica Farrand, Harold Collard

**Affiliations:** 1 University Of California, San Francisco

## Abstract

OBJECTIVES/GOALS: Studying IPF associated health care utilization (HRU) in real world settings, provides the opportunity to produce generalizable results that can directly inform models of care delivery. The objective of this study was to examine real-world differences in the natural history of annual HRU and treatment trends associated with IPF in a large, community-based population of patients with IPF, compared to matched non-IPF controls. METHODS/STUDY POPULATION: Cases of IPF were identified using case validated algorithms in the Kaiser Permanente Northern California adult population from 2000 to 2014. Each case was matched to at least one and no more than five non-IPF controls by age, sex, race/ethnicity and length of enrollment. The date of the first occurrence of the IPF-specific diagnostic code was considered the index date for cases and matched controls. Comorbidity burden and HRU was assessed in the five years pre- and post-index date, including hospitalizations, outpatient visits, use of diagnostic and monitoring studies and medications. Poisson generalized estimating equations models with robust standard errors were used to estimate adjusted case-control differences in HRU, accounting for clustering within matched sets. RESULTS/ANTICIPATED RESULTS: 691 patients were identified with incident IPF during the study period and matched to 3,452 control subjects. Demographics were well balanced between cases and controls due to matching. Patients with IPF had a higher burden of all selected co-morbidities and higher HRU compared to controls. In the pre-index period, IPF members had significantly higher rates of all diagnostic procedures (p < 0.001) and health care visits, with the exception of skilled nursing facility care (p < 0.001). The greatest relative difference was observed with use of Chest CT (RR = 245.94, 95% CI 117.04, 516.82). In the post-index period compared to controls, patients with IPF had higher rates of serial testing (p < 0.001) and inpatient and outpatient care including, all-cause hospitalization (1.55), emergency room visits (1.19), outpatient visits (1.18), and skilled nursing facility stay (1.35). DISCUSSION/SIGNIFICANCE OF IMPACT: Patients with idiopathic pulmonary fibrosis experience increased co-morbidity and healthcare resource utilization compared to controls. This increased burden extends beyond the index-period as previously documented and is true for a large real-world cohort. CONFLICT OF INTEREST DESCRIPTION: NA

